# Functions and Inhibition of Galectin-7, an Emerging Target in Cellular Pathophysiology

**DOI:** 10.3390/biom11111720

**Published:** 2021-11-18

**Authors:** Nishant V. Sewgobind, Sanne Albers, Roland J. Pieters

**Affiliations:** Department of Chemical Biology & Drug Discovery, Utrecht Institute for Pharmaceutical Sciences, Utrecht University, P.O. Box 80082, NL-3508 TB Utrecht, The Netherlands; n.v.sewgobind@uu.nl (N.V.S.); s.l.albers@students.uu.nl (S.A.)

**Keywords:** galectin-7, epithelial tissues, apoptosis, targeting, inhibitors

## Abstract

Galectin-7 is a soluble unglycosylated lectin that is able to bind specifically to β-galactosides. It has been described to be involved in apoptosis, proliferation and differentiation, but also in cell adhesion and migration. Several disorders and diseases are discussed by covering the aforementioned biological processes. Structural features of galectin-7 are discussed as well as targeting the protein intracellularly or extracellularly. The exact molecular mechanisms that lie behind many biological processes involving galectin-7 are not known. It is therefore useful to come up with chemical probes or tools in order to obtain knowledge of the physiological processes. The objective of this review is to summarize the roles and functions of galectin-7 in the human body, providing reasons why it is necessary to design inhibitors for galectin-7, to give the reader structural insights and describe its current inhibitors.

## 1. Introduction: Galectin-7

Galectin-7 belongs to a family of lectins that bind specifically to β-galactosides, i.e., the galectins. To date, 16 different members of galectins have been described in mammals, and 12 members have been characterized in humans. Although galectins share primary structural resemblance in their carbohydrate-recognition domains (CRDs), they are subdivided into three groups based on the molecular architecture. Prototype galectins contain a single CRD and form homodimers (human galectin-1, -2, -7, -10, -13, -14, and -16). Tandem-repeat galectins (human galectin-4, -8, -9, and -12) contain two CRDs that are connected by a short peptide linker that can range from 5 up to 70 amino acids. Finally, there are chimera-type galectins (only member: human galectin-3) when a single CRD is connected to an amino-terminal polypeptide non-lectin domain through which it can form oligomers [1,2,3].

Galectin-7 was first reported by Celis in 1995 while searching for keratinocyte proteins that may play a role in the maintenance of the normal phenotype and various skin diseases. One of these proteins corresponded to IEF17 in the keratinocyte database and had a shared identity with the galectin family. It contained all the amino acids that are central to the β-galactoside binding. For this reason, the protein was named galectin-7 after consultation with researchers in the field [4]. The findings of the Celis group were supported by Magnaldo and colleagues [5]. Both groups concluded that galectin-7 is a keratinocyte-specific marker often found in all layers of the epidermis and other stratified epithelia of tissues; in the tongue, cornea, esophagus, stomach, anus, Hassal’s corpuscles of the thymus and even in myoepithelial cells of the mammary gland epithelium [6].

Galectin-7 is synthesized in the cytoplasm, and it accumulates in the cytosol or nucleus before secretion to the outer plasma membrane or extracellular matrix. Like all other galectins, the secretion or export of galectin-7 from the cytoplasm occurs via an undefined nonclassical secretory mechanism [1,7].

The X-ray crystal structure of human galectin-7 in its native form is described by the Celis and Acharya groups as a dimer. It has a significant amino acid sequence identity to the known prototype of galectin-1, -2 and -10 [8]. Although it was reported as a monomer in solution [8,9], the observed molecular weight as determined by ultracentrifugation and sedimentation experiments strongly suggests that it is a dimer in solution [10]. Nesmelova and co-workers confirmed these findings and reported ^1^H, ^13^C, and ^15^N chemical shift assignments for the human galectin-7 dimer as determined by heteronuclear, triple resonance NMR spectroscopy in solution [11].

Acting intra- or extracellularly, galectin-7 participates in diverse processes, such as controlling apoptosis, cell migration and cell adhesion. In addition, it also plays a crucial role in the re-epithelialization process of corneal or epidermal wounds and in several human diseases/disorders, such as cancer [12]. Because of its diverse roles in human cellular pathology and the fact that the precise modes of action of galectin-7 are not well understood in many cases, there is a need for strong inhibitors that target galectin-7 specifically in order to provide insights into the biological mechanisms and as a string point for therapeutic intervention. This review intends to present an up-to-date overview on galectin-7 and its various roles in the human body from a chemical as well as a biological point of view. We aim to do this by covering the following subjects: biological importance of galectin-7, targeting galectin-7, and structural features. We will refer to the current synthetic inhibitors of galectin-7.

## 2. Galectin-7, a Convergence of Pathology with Physiology

Being mainly expressed in stratified epithelia, galectin-7 is described in epithelial tissues as being involved in apoptotic responses, proliferation and differentiation, but also in cell adhesion and migration [13]. In the following section, we will examine its involvement by elaborating on several biological processes and disorders which are linked to (the functions of) galectin-7.

### 2.1. Role in Epidermal Homeostasis of Skin, Corneal and Periodontal Tissue

Bernerd et al. showed that UVB irradiation of skin keratinocytes, reconstructed in vitro and of human skin *ex vivo*, lead to sunburn/apoptotic skin keratinocytes. These sunburn/apoptotic keratinocytes express higher levels of galectin-7 than other keratinocytes, suggesting that galectin-7 is strongly associated with UVB-induced apoptosis in the epidermis [14].

The previously obtained result by Bernerd et al. was confirmed and revealed that the expression of galectin-7 is induced by UVB irradiation and also *cis*-UCA (*cis*-urocanic acid). The latter is an epidermal chromophore that undergoes *trans* to *cis* isomerization after UVB irradiation. Notably, *cis*-UCA is a potent inhibitor of cutaneous acquired immunity. It was concluded that galectin-7 induces apoptosis and demonstrated that it is highly expressed in the epidermis of patients with actinic keratosis, compared with normal skin [15].

Gendronneau et al. found evidence for the role of galectin-7 in the process of skin wound healing. They generated galectin-7–deficient mice that were viable and exhibited no phenotypical abnormalities in skin structure, organization, differentiation or expression of epidermal markers. However, the epidermal response to UVB radiation as well as mechanical injury in vivo proved to be disturbed. Sunburn cells occurred earlier, the apoptotic response was less acute, and it lasted longer, compared with wt (wild-type) tissue. It was concluded that galectin-7 modulates keratinocyte apoptosis and proliferation as well as migration [16].

In addition, the same group studied the role of galectin-7 overexpression in basal keratinocytes of skin repair after environmental stress. The epidermal response to a scratch on the surface was delayed (timing of wound closure). The re-epithelialization of cells located at each edge of the wound depends on cellular interactions, notably through adherens junctions. It was proposed that the overexpression of galectin-7 causes the loosening of adherens junctions and hence, the delay in wound closure. When the transgenic mice (with keratinocytes overexpressing galectin-7) were exposed to UVB radiation, more keratinocyte apoptosis was induced. The effects on the maintenance of epidermal homeostasis of deficient and overexpressed galectin-7 were proven to be very similar [17]. Advedissian and co-workers continued the study of the involvement of galectin-7 in cell migration and found that there is an interaction with a key component of adherens junctions, E-cadherin. They showed an interaction between galectin-7 and E-cadherin at the plasma membrane, which causes intercellular adhesion [18].

Mechanistic evidence was provided for the aforementioned findings of Gendronneau et al. The galectin-7 knockdown results in reduced differentiation and increased proliferation of keratinocytes. Moreover, it was shown that galectin-7 positively regulates microRNA (miR)-203 expression, which in turn is used for regulating keratinocyte differentiation and proliferation. To determine how galectin-7 regulates keratinocyte proliferation and differentiation through miR-203, the expression of a known miR-203 target, p63 (an essential transcription factor involved in skin development), in galectin-7 knockdown cells was examined. Knocking down either galectin-7 or miR-203 in keratinocytes increased the expression of p63. The rescue of miR-203 expression in a galectin-7 knockdown model reduced p63 expression. Further extensive research showed that increased galectin-7 expression upregulates c-Jun N-terminal kinase 1 (JNK1) by a direct interaction, which is required for miR-203 expression. Finally, they established that galectin-7 has an intracellular function in keratinocytes through the JNK1-miR-203-p63 pathway [19]. More recently, it was found that the expression of galectin-7 is reduced by cytokines in the skin lesions of patients with psoriasis. This results in the hyperproliferation of keratinocytes and skin inflammation [20].

Systemic sclerosis (SSc) is a multisystem connective tissue disorder characterized by vascular injury, fibrosis of the skin, various internal organs following autoimmune inflammation and tissue injury [21]. Saigusa and co-workers investigated the potential contribution of galectin-7 to the development of clinical manifestations in SSc, using clinical samples from patients and cultured keratinocytes. Galectin-7 proved to be remarkably downregulated in the basal and suprabasal layers of the lesional epidermis of involved skin in contrast to the abundant expression throughout the epidermis of normal control skin. In addition, SSc patients with diffuse pigmentation and those with esophageal dysfunction had significantly decreased serum galectin-7 levels as compared to those without each symptom. Suppression of the galectin-7 level is believed to be stimulated by autocrine endothelin signaling stimulation in SSc keratinocytes [22].

Patients who suffer from diabetes mellitus [23] have a high risk of impaired wound healing that sometimes may lead to infection and amputation. As cell migration is an important process involved in proper wound healing, Huang and co-workers demonstrated that a high glucose environment reduced galectin-7 expression in keratinocytes, due to enhanced *O*-GlcNAc (*O*-linked *N*-acetyl-D-glucosamine) glycosylation of certain regulators of galectin-7 expression. This dysregulation of galectin-7 causes a significant reduction of keratinocyte migration and thus, improper wound healing [24]. The context of this dysregulation can be associated with *O*-GlcNAc-mediated processes controlling cellular differentiation [25]. A more detailed review regarding the re-epithelialization of skin wounds is reported [26].

In their search for novel, galectin-based therapeutic strategies for the treatment of nonhealing corneal tissue epithelial defects, Cao et al. demonstrated via Western blot analysis that healing corneas contained increased levels of galectin-7 throughout the epithelium, compared with normal corneas after injury. Furthermore, it was reported that exogenous galectin-7 stimulated the rate of corneal epithelial wound closure. Inhibition of this stimulatory effect of galectin-7 occurred by a competing lactose but not by non-binding sucrose. It was suggested that the CRD of the lectin is directly involved in wound closure [27].

Er: YAG (erbium-doped yttrium–aluminum–garnet) laser therapy is used for periodontal treatment by removing soft and hard tissues as well as calculus, with minimal heat-related side effects. The bactericidal effect makes the therapy even more useful. Er: YAG laser irradiation promotes faster adhesion and growth of human gingival fibroblasts (HGFs) and periodontal ligament fibroblasts (PDL fibroblasts). The cell proliferation of HGFs is reported to be stimulated, and this might be caused due to an increase in the protein expression of galectin-7 in the HGFs. Er: YAG laser irradiation causes a direct effect of promoting proliferation, migration, and invasion of PDL fibroblasts through the upregulation of galectin-7, yet its signaling pathway needs to be verified [28].

### 2.2. Roles in Cancer

Approximately 85% of cancers occur in epithelial cells: the carcinomas [29]. Like many galectins, galectin-7 displays opposite effects in terms of tumor progression from one histological type to another. It may contribute to the growth and/or development of certain tumor types, while acting negatively on the development of other tumor types [12]. Galectin-7 does not only have a role in carcinomas [13], but also in lymphomas and melanomas by contributing either to neoplastic transformation and tumor progression through the regulation of cell growth, cell cycle, angiogenesis, apoptosis and cell migration. In addition, galectin-7 may have a protective effect on cancer, depending on the tissue type [30]. Hanahan and Weinberg defined hallmarks of most cancers which describe the biological capabilities essential for carcinogenesis [31]. There are a number of papers published regarding the subject of cancer (development) and the roles of (targeting) galectins, and even galectin-7 in particular [13,30,32,33,34,35,36,37,38,39,40,41,42,43,44,45,46,47,48,49,50]. Nevertheless, our goal for this section is to provide the reader with a brief overview of the presence and roles of galectin-7 in most cancers/cancer types by covering mostly recent publications.

Analysis of the expression of galectin-7 in benign and malignant thyroid cancers showed a downregulation of galectin-7 in adenomas, compared to carcinomas [51]. It was shown that galectin-7 is constitutively expressed in aggressive (metastatic) lymphoma cells at both mRNA and protein levels. Highly metastatic variants of the lymphoma cell line showed strong upregulation of galectin-7 in the spleen, the thymus and kidneys, due to the methylation of the galectin-7 gene (*LGALS7*) [52,53]. Methylation of the *LGALS7* gene, leading to the silencing of galectin-7 during gastric cancer tumorigenesis, was also suggested by Kim and colleagues. They revealed significantly lower expression levels of galectin-7 in malignant tissues of gastric cancer patients, compared with matched normal tissues. The overexpression of galectin-7 in AGS gastric adenocarcinoma cells suppressed cell proliferation, migration, and invasion, whereas the removal of galectin-7 in KATO III gastric carcinoma cells reversed these properties [54].

To determine its critical role in lymphoma progression, Demers and co-workers hypothesized two years later that the promalignant activity of galectin-7 in thymic lymphoma is related to its capacity to induce MMP-9 (matrix metalloproteinase-9, a metastatic gene) expression. Their hypothesis was based on the evidence that galectin-7 transfectants have higher levels of MMP-9 expression, while the addition of lactose completely inhibits the expression of MMP-9. Furthermore, murine or human recombinant galectin-7 induces the expression of MMP-9 in both mouse and human lymphoma cells [55]. In continuation, the same group found evidence that galectin-7 is expressed in human lymphoid malignancies and proposed that it is a critical tumor-modulating gene that controls the dissemination of lymphoma cells via MMP-9 [56]. The reader is also referred to the review by St-Pierre regarding the relationships between galectin-7, p53 and MMP-9 during cancer progression [6].

Galectin-7 was reported to be highly expressed in ESCC (esophageal squamous cell carcinoma) during a study that was designed to isolate and identify ESCC biomarkers, using proteomic tools. The level of galectin-7 expression was related to the degree of ESCC differentiation [57].

Galectin-7 is also believed to increase the invasive behavior of breast cancer cells; the ability to metastasize to the lungs and bones increased in mouse models. It is believed that breast cancer cells overexpressing galectin-7 are related to the ability of galectin-7 to protect against apoptosis [58]. An important mediator of galectin-7 gene activation in breast cancer cells, CCAAT/enhancer-binding protein beta or C/EBPβ, was suggested to contribute by the same group in 2014 [59]. Grosset et al. generated a mutant form of galectin-7 in which arginine 74 was mutated to obtain galectin-7^R74S^, a CRD-defective mutant form of galectin-7. They demonstrated that breast cancer cells expressing mutated galectin-7 were equally or even more resistant to drug-induced apoptosis, compared to cells expressing wt galectin-7 [60]. In addition, galectin-7 proved to accelerate tumor progression in one of the most aggressive forms of breast cancer (HER-2 positive) as was published in a subsequent study, using genetically engineered galectin-7–deficient mice [61].

The observation that galectin-7 may have immunosuppressive properties was made by Labrie and co-workers while investigating the expression of galectin-7 in epithelial ovarian cancer (EOC). It was found that galectin-7 increased the invasive behavior of ovarian cancer cells by inducing MMP-9 and increasing cell motility. EOC cells can also secrete galectin-7. Recombinant human galectin-7 kills Jurkat T cells and human peripheral T cells [62].

In contrast, galectin-7 reduces the invasive behaviors of prostate cancer cells by inhibiting their motility. Galectin-7 is found to be downregulated in prostate cancer cells, and the expression of galectin-7 in prostate cancer cells increases their sensitivity to apoptosis in response to chemotherapeutic agents. The group of St-Pierre showed that the ability of galectin-7 to modulate apoptosis was independent of its CRD activity by using a CRD-defective mutant, i.e., galectin-7^R74S^. However, CRD activity proved to be necessary to inhibit the invasive behaviors of prostate cancer cells. In vivo, galectin-7 overexpression in prostate cancer cells led to a significant reduction in tumor size, while its CRD-defective mutant form significantly increased tumor growth [63].

The group of Lo demonstrated that human tumorous imaginal disc (Tid1), a heat shock protein (Hsp40), reduces head and neck squamous cell carcinoma (HNSCC) malignancy. It was found that galectin-7 was one of the proteins that interact with Tid1 and the levels of expression of both proteins were measured in HNSCC patients. Low Tid1 and high galectin-7 expression predicted poor overall survival in HNSCC. The interaction between Tid1 and galectin-7 was bridged by *N*-linked glycosylated Tid1. It is believed that *N*-linked glycosylation of Tid1 is required to interact with galectin-7 to downregulate galectin-7, which in turn can attenuate cancer progression and metastasis. Galectin-7 played a critical role in promoting tumorigenesis and metastatic progression by enhancing the transcriptional activity of TCF3 transcription factor through elevating MMP-9 expression [64].

Evidence was provided for the pro-invasive activity of galectin-7 in oral squamous cell carcinoma (OSCC) by inducing the expression of not only MMP-9, but also MMP-2. It was observed that galectin-7 overexpression resulted in significant upregulation of MMP-2 and MMP-9. On the other hand, silencing MMP-2 or MMP-9 significantly impaired the invasiveness of OSCC cells that overexpressed galectin-7. In order to explain these results, the signaling pathways involved were investigated. It was concluded that increasing galectin-7 expression significantly enhanced the phosphorylation and activation of extracellular signal-related kinase (ERK) and c-Jun N-terminal kinase (JNK). Moreover, the pharmacological inhibition of ERK or JNK activity significantly reduced OSCC cell invasiveness induced by galectin-7 overexpression [65]. The signaling pathways which direct hypersensitized carcinoma cells to apoptosis was also earlier observed in malignant peripheral nerve sheath tumor cells [66].

The proapoptotic activity of galectin-7 was also attributed to activation of the JNK pathway in cervical and colon cancer [67,68]. Zhu and colleagues confirmed these results by revealing a role for galectin-7 in sensitizing cervical squamous cancer cells to paclitaxel treatment. A galectin-7 knockdown in the cancer cells showed increased viability against paclitaxel-induced apoptosis [69]. As galectin-7 is negatively regulated in cervical cancer, Higareda-Almaraz and co-workers demonstrated the link between the pro-apoptotic response triggered by cancer and the anti-tumoral activity of the immune system. Galectin-7 re-expression affects the regulation of molecular networks in cervical cancer that are involved in some of the cancer hallmarks, such as metabolism, growth control, invasion and evasion of apoptosis. The effect of galectin-7 extends to the microenvironment, where the reconstitution of galectin-7 leads to a change of regulation and interaction networks [70].

It was demonstrated by Menkhorst and colleagues that galectin-7 production increased in endometrial cancer with increasing cancer grade; galectin-7 may promote the metastasis of endometrial cancer by reducing cell–cell adhesion and enhancing cell migration. Furthermore, it was also established that galectin-7 had no significant effect on proliferation or apoptosis [71].

Matsui and co-workers showed that bladder cancer cells expressing upregulated galectin-7 tended to respond more sensitively to chemotherapy, compared to urothelial tumor cells having lower levels of galectin-7 [72].

Kopitz et al. demonstrated for human neuroblastoma cells that galectin-7 is a negative growth regulator not by apoptosis, but rather a switch from proliferation to differentiation of the cancer cells [73].

### 2.3. Role in Pre-Eclampsia, Menstruation and Recurrent Pregnancy Loss

Pre-eclampsia is a hypertensive disorder of pregnancy and causes maternal and fetal morbidity and mortality. It is defined as the presence of hypertension, proteinuria or other end organs, such as liver or brain, damage occurring after 20 weeks of pregnancy. Severe forms of pre-eclampsia can be complicated by renal, cardiac, pulmonary, hepatic, and neurological dysfunction, hematologic disturbances, fetal growth restriction, stillbirth and maternal death [74,75]. Recurrent pregnancy loss is a prevalent and distressing disorder, defined as the spontaneous end of pregnancy before an embryo has reached viability until 20–24 weeks of gestation [76,77].

Members of the galectin family are expressed within the female reproductive tract and have been shown to be involved in multiple biological functions that support the progression and regulation of implantation and pregnancy via cell adhesion and migration, immune cell activation, apoptosis and hormone production to name a few [78,79].

Menkhorst et al. investigated the expression of galectin-7 in the endometrium during the menstrual cycle of normally fertile women and women who have a history of miscarriage to see whether there is an association with tissue/serum levels of galectin-7 and miscarriage. Galectin-7 was immunolocalized to the endometrial luminal and glandular epithelium in normally fertile women. The serum concentration of galectin-7 proved to be significantly elevated at week 6 of gestation in women with a viable fetus with a history of miscarriage, compared to normal healthy pregnancies. Furthermore, galectin-7 was aberrantly expressed in the non-pregnant endometrium of women with a history of miscarriage. These findings suggested that this allows for inappropriate blastocyst implantation. They demonstrated a role for galectin-7 on trophoblast–endometrial epithelial cell adhesion by acting as an adhesion molecule [80]. In a subsequent study, the same group showed that galectin-7 serum concentration was significantly elevated during weeks 10–12 and 17–20 of gestation in women who went on to develop pre-eclampsia, compared to women with normal pregnancies. It was also proposed that the elevated serum galectin-7 associated with pre-eclampsia may be due to placental oxidative stress and/or hypomethylation [81].

Evans and colleagues were able to identify and compare endometrial expression of galectin-7 in women with normal endometrial repair versus women with amenorrhea who do not experience endometrial breakdown and repair. Their study demonstrated the presence of galectin-7 not only within the menstruating endometrium (being produced by the premenstrual and menstrual endometrium), but also in menstrual fluid. They also established that galectin-7 enhances endometrial re-epithelialization and elucidated the mechanism by which galectin-7 mediates endometrial epithelial wound repair. Galectin-7–mediated re-epithelialization is dependent on integrin-mediated signaling and elevates the expression of ECM factors which are involved in repair in other tissues [80].

In order to study the function of (among other) prototype galectins in placental tissue, the expression of galectin-7 in the placenta in cases of spontaneous abortions (SPA) and recurrent abortions (RA) in the first trimester was analyzed. Galectin-7 was found in the syncytiotrophoblast in placentas after induced abortion and with weaker staining in the decidua. In SPA and RA first-trimester placentas, the expression of galectin-7 in the villous trophoblast/syncytiotrophoblast was significantly lower [82].

In order to determine the role of galectin-7 in the placenta, Menkhorst and co-workers demonstrated that elevated galectin-7 during placental formation contributes to abnormal placentation, thus leading to the development of pre-eclampsia. Augmented galectin-7 during the period of placental formation in mice caused hypertension and albuminuria, and the authors hypothesize that in women, galectin-7 acts via the placenta to induce the systemic features of pre-eclampsia via impaired placental formation, placental inflammation and the placental release of anti-angiogenic factors [83].

### 2.4. Roles in Allergic Inflammatory and Autoimmune Diseases

Inflammatory autoimmune diseases have large numbers of pathologies characterized by various factors that can contribute to a breakdown in self-tolerance or inflammation dysregulation. Immune cells are sensitive to galectins, and they are important regulators of inflammation or autoimmunity, making them therapeutic targets for some inflammatory autoimmune diseases [84].

Galectins control a wide range of cells involved in the allergic inflammatory diseases by modulating the biological activities of the cells. Hence, galectins may influence the development and course of allergic diseases. Evidence for the involvement of galectins in terms of immunoregulatory activities has been gathered in the pathogenesis of allergic conjunctivitis, atopic dermatitis, asthma and food allergy in the past few years [85].

During their study, Niiyama and colleagues assessed whether galectin-7 could be utilized as an indicator (biomarker) of skin barrier disruption and as an index of local skin symptoms in atopic dermatitis (AD) patients. Atopic dermatitis is a chronic, relapsing inflammatory skin disease characterized by pruritic and eczematous skin lesions. Skin barrier disruption is an important contributing factor in the pathogenesis of AD, as the disruption of the skin barrier allows the penetration of allergens into dry skin, inducing an itching sensation. Galectin-7 expression in keratinocytes increased after skin barrier disruption, and an overexpression in the stratum corneum was detected in tape-stripped samples. Measurement of the galectin-7 content in the stratum corneum might be useful for the evaluation of the skin barrier function in dry skin conditions, such as AD [86].

Niiyama’s results were confirmed, and the production mechanism and functional role of galectin-7 in AD patients was investigated. A galectin-7 knockdown experiment on a 3D-reconstructed epidermis was performed; it resulted that endogenous galectin-7 protects IL-4/IL-13–induced disruption of cell-to-cell adhesion and/or cell-to-extracellular matrix adhesion. In addition, IL-4/IL-13–induced galectin-7 release from keratinocytes reflects the skin barrier impairment in AD patients [87].

Luo and co-workers showed that galectin-7 promotes activated CD4+ T cell immunity. The modes of action include the promotion, proliferation and polarization of Th1/2 cells balance toward Th1 in activated CD4+ T cells, and the elevation of immune-enhancement factors in the microenvironment by inhibiting the TGFβ/Smad3 pathway. This means that galectin-7 may have anti-inflammation effects, and it can induce autoimmune disease and transplantation rejection [88].

The airway epithelium plays an important role in the development of allergic inflammation, remodeling, and bronchial hyper-responsiveness. Moreover, the bronchial epithelium plays an important role in immune regulation during the initiation of allergic responses. The integrity of airway epithelial layer structure is the key to the airway barrier and local microenvironment homeostasis. Destruction of the integrity of the epithelium leads to depletion of the ordered airway barrier and increases sensitivity to viral infections and allergens. Eventually, this leads to airway inflammations, such as asthma or chronic obstructive pulmonary disease (COPD) [89].

As galectin-7 was identified to be overexpressed and increased apoptosis occurred in bronchial epithelial cells in asthma, Sun and Zhang investigated the effect of galectin-7 on the apoptosis of human bronchial epithelial cells. They were able to demonstrate that galectin-7 silencing inhibited TGF-β1–induced (growth factor that promotes multiple cell apoptosis, also elevated in asthmatic patients) apoptosis in airway epithelial cells via blocking the JNK pathway [90].

Encouraged by their previously obtained results, Tian et al. showed that the expression of galectin-7 mRNA and protein in bronchial epithelial cells of children with asthma were both increased, and the expression of galectin-7 mainly occurred in apoptotic bronchial epithelial cells. The overexpression of galectin-7 in transgenic mice (Tg(+) mice) showed abnormal airway structures in embryos and after birth; a thin and disordered epithelium layer was observed. Galectin-7 was localized in the cytoplasm and nucleus of bronchial epithelial cells. Increased apoptosis was mediated through the mitochondrial release of cytochrome c; upregulated JNK1 activation and expression destroys the airway epithelium barrier, which predisposes the airways to RSV respiratory syncytial virus (RSV), ovalbumin or OVA-induced epithelial apoptosis. Taken together, the aforementioned results suggest that galectin-7 causes airway structural defects, injury, and other asthma responses [89].

Intracellular galectin-7 proved to be involved in bacterial autophagy, as immunoblotting analysis by the group of Lin and co-workers revealed low-level galectin-7 expression in HeLa cells. Examination of HaCaT cells revealed that intracellular galectin-7 clearly colocalized with and surrounded group A streptococcus (GAS), an intracellular bacterium. GAS proliferation was increased following galectin-7 knockdown in HaCaT cells, which indicates that intracellular galectin-7 plays a critical role in intracellular immunity in the response against bacterial infection [91].

### 2.5. Role in Transplant Rejection

Based on the facts that galectin-7 is related to immune responses in transplantation and increased expression of galectin-7 in serum from renal allograft recipients (compared with normal volunteers) was identified, Luo and colleagues investigated the galectin-7 response to acute rejection of mouse cardiac allografts. More specifically, they showed that the expression of galectin-7 increased with the severity of allograft rejection. Furthermore, they demonstrated that the upregulation of galectin-7 expression in the allografts was directly related to the T cell response. The results showed that infiltrating lymphocytes and endothelial cells in the allografts expressed large amounts of galectin-7 located in the cytoplasm and nucleus of cardiomyocytes, endothelial cells, and infiltrating lymphocytes. This was not observed in native hearts or isografts, and it is believed that galectin-7 plays a crucial role to accelerate allograft rejection [92].

Table 1 summarizes the various pathophysiological roles and mode of action displayed by galectin-7.

Despite of the many findings mentioned in this section, much has to be discovered at the molecular level of several pathophysiological processes. Knocking down or not expressing galectin-7 would not be sufficient in many cases, and hence it may cause any other complications.

## 3. Drug Potential of Galectin-7 Inhibitors and Galectin-7 as a Biomarker

It may not always be necessary to solely inhibit galectin-7 either intracellularly of extracellularly. Clearly, inhibitors will eventually aid the elucidation of molecular mechanisms/pathways in a variety of biological processes, but it is also of great interest to support the diagnosis and prognosis of several disorders. The second part of this section will deal with the use of galectin-7 as a biomarker in some cases where it is reported to be overexpressed.

### 3.1. Drug Potential of Galectin-7 Inhibitors

The activity and function of any galectin can be multi-faceted, due to galectin self-association, and/or interactions with cell surface glycans/other biomolecules, both extracellularly and intracellularly [93].

The approach of carbohydrate-derived small-molecule inhibitors to target the CRD of galectins is mainly based on the use of chemically modified natural galectin ligands, such as the disaccharides lactose (Lac) or *N*-acetyllactosamine (LacNAc). As the development of these inhibitors involves a full understanding of the biochemistry of galectin–glycan interactions, efforts are being made to generate galectin inhibitors that target individual members (particularly galectins-1, -3 and -7) of the family with higher affinity and selectivity [50]. Most of the current inhibitors only block extracellular functions of a given galectin and neglect intracellular functions [36], except for galectin-3 for which Stegmayr and co-workers were able to synthesize and evaluate the roles of intracellular and extracellular galectin-3 inhibitors [94].

It is warranted in impaired diabetic wound healing to identify and elucidate the status of specific galectin-7 regulating molecules in a high glucose environment. Furthermore, elucidating the specific molecular dysfunction in keratinocytes associated with individual diabetic phenotype will likely result in the development of more effective and personalized therapeutic strategies for optimal wound management in patients diagnosed with diabetes [24].

Wan and colleagues concluded in their review that inhibiting the contribution of galectin-7 to allergic inflammation should be achievable by generating antibodies with the proviso that (1) the antibodies do not exhibit cross reactivities to other galectins and (2) the galectin’s contribution should go through extracellular actions. If this is not the case, antibodies will not be suitable, and cell-permeable inhibitors are required [85].

As for the resistance to anticancer therapies, intracellular versus extracellular functions of galectins are an important aspect to keep in mind to understand the role of these proteins in anticancer therapy resistance, as well as in the design of galectin-based cancer treatments [48].

Many publications call for inhibitors and methods for targeting galectin-7 and/or modulating its activity. Yet, no specific galectin-7 inhibitor is available. For example, extracellular galectin-7 promotes cancer via binding to cell surface receptors of cancer cells and induce *de novo* transcriptional activation of *LGALS7*, which in turn render cells resistant to pro-apoptotic drugs. Another example is displayed by the binding of extracellular galectin-7 to glycoreceptors expressed in infiltrated immune cells that triggers a cascade of signaling events, leading to the apoptosis of cancer-killing T cells, or alters their regulatory functions, helping tumors evade anti-tumor immunity [95].

In another example, where the expression of galectin-7 in epithelial ovarian cancer (EOC) is evaluated, it was observed that extracellular galectin-7 is released outside the cells. Galectin-7 is believed to have a significant impact on tumor progression by inducing immunosuppression and increasing the invasive behavior of tumor cells that eventually leads to metastasis. Targeting galectin-7 may represent a valuable strategy to overcome cancer-associated immunosuppression and the prevention of metastasis in EOC [62].

Grosset and co-workers confirmed the expression of galectin-7 in the cytosolic and nuclear compartments of breast cancer cells and the ability of galectin-7 to translocate to mitochondria. However, whether the resistance of breast cancer cells to apoptosis is dependent on the intracellular localization of galectin-7 remains unknown [60,96]. Bibens-Laulan and St-Pierre uncovered how galectin-7 traffics between both intracellular and extracellular compartments in ovarian and breast cancer cells. They reported that extracellular galectin-7 plays a central role in controlling intracellular galectin-7 in cells via two mechanisms: firstly, by increasing the transcriptional activation of *LGALS7* gene transcription, and secondly via re-entry into the cells. However, whether re-entry is dependent on the glycan-binding site of galectin-7 is unknown [97]. Girotti and colleagues concluded that it is still not clear whether intracellular or extracellular activities of galectins should be targeted to halt tumor progression [49]. In addition to their extracellular function, the fact that galectins can alter tumor progression through their interaction with intracellular ligands (sometimes even independently of their CRD) calls for a change in the basic assumptions and may force scientists re-design strategies in order to develop galectin antagonists for the treatment of cancer [98].

### 3.2. Galectin-7 as a Biomarker

The biomarkers field is shifting from tests analyzing single targets to multiplexed analysis of numerous proteins with or without post-translational modifications or exclusively glycans. These improvements are possible, due to the advances in analytical (detection) techniques, such as mass spectrometry for glycan analyses and lectin-antibody array methodologies. Indeed, a more specific (and early) pathological (i.e., cancer) diagnosis will result in earlier disease detection, improved disease monitoring and assistance and eventually successful patient-specific therapies. However, despite all the literature supporting the value of biomarkers for prognostic and monitoring applications, these tests suffer from limited specificity and sensitivity, which makes it a challenge to come up with a useful biomarker [38,41].

Stevens–Johnson syndrome (SJS) and toxic epidermal necrolysis (TEN) are severe cutaneous adverse drug reactions (cADRs) that can cause a life-threatening condition and late sequelae. Galectin-7 was reported to be one of the seven proteins that showed higher concentrations in the samples of SJS/TEN samples than in the non-severe cADR samples. The proteins were quantitated, using selected/multiple reaction monitoring (SRM/MRM) with stable synthetic isotope-labeled peptides as an internal control. The technique might be useful in the search for a potential SJS/TEN biomarker and key candidates involved in SJS/TEN pathogenesis [99].

Although it was proposed that galectin-7 serves as a negative prognostic factor in ovarian cancer by two independent groups [62,100], Schulz and colleagues studied the prognostic value of galectin-7 (among other galectins) in patients with epithelial ovarian cancer. The staining of galectin-7 in tumor cells was mainly observed in the cytoplasm; only a few individual cases showed nuclear staining. In addition, a significantly reduced overall survival was observed for cases with a high galectin-7 expression and a better survival for galectin-7 negative cases. Lower galectin-7 expression was confirmed as an independent prognostic factor for overall survival in ovarian cancer [101].

Trebo et al. suggested that galectin-7 might be an independent negative prognostic factor in breast cancer and a therapeutic target, especially in HER2-positive breast cancer. The expression of galectin-7 was observed in the cytoplasm as well as in the nucleus of breast cancer cells. Galectin-7 expression in the cytoplasm as well as in the nucleus was significantly higher in no special type (NST) tumors, compared to non-NST tumors. In addition, galectin-7 was also present in macrophages next to the tumor cells. These macrophages might also provide a source of extracellular galectin-7 for tumor cells and might regulate the intracellular galectin-7 pool. Combining the results suggested that galectin-7 might be an independent negative prognostic factor in breast cancer and a therapeutic target, especially in HER2-positive breast cancer [102].

Matsukawa and co-workers aimed to identify predictors of tumor sensitivity to preoperative radiotherapy/chemotherapy for oral squamous cell carcinoma (OSCC) in order to allow oncologists to determine optimum therapeutic strategies. They identified galectin-7 as a potential predictive marker of chemotherapy and/or radiotherapy resistance, as in vitro overexpression of galectin-7 significantly decreased cell viability after chemotherapy (most likely due to growth arrest rather than apoptosis) in the OSCC cell line [103].

Kim et al. indicated that, given the fact that the expression of galectin-7 in gastric cancer is regulated by DNA hypermethylation (as discussed previously in Section 2), the DNA methylation of galectin-7 is a promising candidate biomarker for application in gastric cancer [54].

In order to develop new inhibitors for galectin-7, one must gain knowledge regarding structural information of the binding pocket and to have a understanding of the preferred interactions between the target protein and small molecules. The following section will cover these aspects.

## 4. Structural Features

Being involved in a variety of physiological processes, many of which are directly linked to immunity and disease, deciphering the complex structures of galectins and their interactions with carbohydrates is of fundamental relevance to gain a deeper understanding of the underlying biological processes involved, the different affinities for different carbohydrates and non-carbohydrate ligands and to develop potential therapeutic interventions [104]. The crystal structures of most of the galectins, also in complexes with glycan ligands, are known. The CRD (consisting of ~130–140 residues) of most galectins is comprised of five- and six-stranded anti-parallel β-sheets arranged in a β-sandwich (sometimes referred to as “jelly roll”) configuration that lacks an α-helix. The subunits in the dimeric galectin-7 are related by a twofold rotational axis perpendicular to the plane of the β-sheets [1].

The first crystal structures of human galectin-7, in free form and in the presence of galactose, galactosamine, lactose, and *N*-acetyl-lactosamine, were published by Leonidas et al. The structure of galectin-7 shows a fold similar to that of prototypes galectin-1 and -2, but has a greater similarity to the related galectin-10. Unlike galectin-1 and -2 that are both dimeric galectins with a single CRD and both known for their multivalent carbohydrate recognition due to their structural organization, the homodimer arrangement of galectin-7 is considerably different because this galectin recognizes carbohydrates in its monomeric form and does not possess multivalency. The dimer interface involves the association of the β-strands, F1–F5, from the two protomers which are held together by hydrogen bonding interactions. These H-bonds involve five residues from molecule (subunit) A, eight residues from molecule (subunit) B, and an extensive set of van der Waals interactions. The dimer interface of galectin-7 is relatively large, 1484 Å^2^, compared to areas of 1093 Å^2^ (galectin-1) and 1179 Å^2^ (galectin-2) [8].

**Figure 1:**
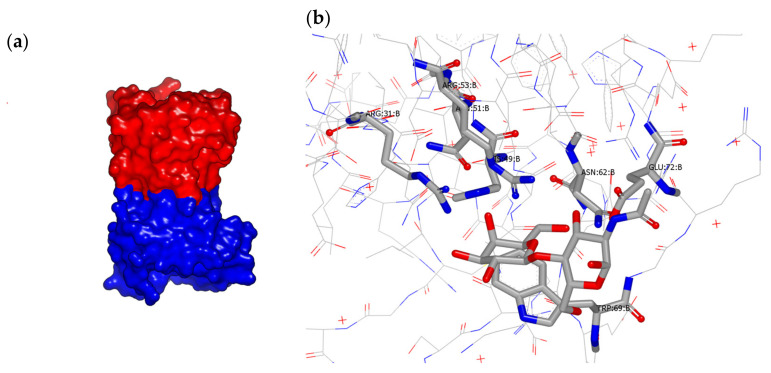
(**a**) Dimeric structure of galectin-7 (pdb 1BKZ); (**b**) *N*-Ac-LacNAc binding to galectin-7 (pdb 5GAL).

Detailed analysis of the aforementioned galectin-7–carbohydrate complex structures show that His49, Asn51, Arg53, Asn62 and Glu72 are the key residues involved in carbohydrate recognition through hydrogen bond interactions. The highly conserved residues His49, Asn 51, and Arg53 make hydrogen bonds with the galactose O4 in all four complexes. The galactose O5 makes two hydrogen bonds with Arg53 and Glu72, while O6 is engaged in interactions with Asn62 and Glu72. Tryptophan 69 is involved in stacking interactions with the galactose moiety in a manner analogous to that seen in Gal-1 and Gal-2 structures. Residues Arg 53, Thr56, Glu58, Glu72, and Arg74 form a network of ionic interactions. In the galactose and galactosamine complex structures, the O1 (involved in hydrogen bond formation with Pro85 and Ser8), O2, and O3 atoms of the carbohydrate are involved in water-mediated interactions and contribute to the strength of carbohydrate binding. Moreover, the Arg31 residue in galectin-7 could form part of the carbohydrate-binding region, as it was observed that Arg31 in galectin-7 occupies the position of His52 in Gal-1, which is located about 3.1 Å away from the carbohydrate moiety [8]. Figure 1 displays the dimeric structure of galectin-7 as well as its binding to *N*-Ac-LacNac.

By combining nuclear magnetic resonance (NMR) and circular dichroism spectroscopies and molecular dynamics (MD) simulations, Ermakova et al. provided complementary structural information on the binding of lactose to galectin-7 and its impact on protein thermodynamics and conformational dynamics. They were able to show that there is positive cooperativity when lactose binds. Binding of the first ligand enhances binding of the second. Analyzing MD simulations indicated that significant changes occur in the ligand-free subunit (A) when lactose is bound to the other subunit (B). Increased conformational entropy was reflected by an overall increased internal motion in galectin-7. Based on molecular mechanics and MD simulations, an increase in the ligand binding–induced dimer stability (of galectin-7) was observed. This increase was validated experimentally in several assays: gel filtration fast protein liquid chromatography (FPLC), CD-based thermal denaturation studies, fluorescence resonance energy transfer (FRET) and STD NMR. Furthermore, it was observed that the binding of lactose to galectin-7 (K_d_ = 0.465 mM averaged over two K_a_ values) alters the lectin conformation and dynamics within the ligand-binding site, as well as through an internal gradient from the ligand-binding site to the dimer interface. The greatest effects were observed in the residues that interact directly with the ligand (the 50–58 and 62–70 loops), the 5-stranded β-sheet at the backside of the lactose-binding site (including the region involved in dimerization of galectin-7) and loops (residues 9–14 and 110–116) down to the dimer interface [105].

**Figure 2:**
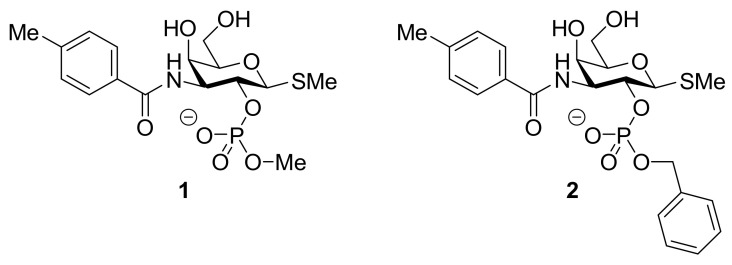
Structures of **1** and **2**: these two molecules differ by the presence of the *O*-benzylphosphate group in **2,** compared with an *O*-methylphosphate group in **1**.

Masuyer and colleagues compared the binding affinities of compounds **1** and **2** (Figure 2) and evaluated the structural information by measuring a high resolution crystal structure of galectin-7 in the complex with **2** [106].

They reported that the CRD itself remains unchanged despite a slight movement in the adjacent loop composed of Arg74 and Gly75. The crystal structure highlights stronger binding achieved through the side groups of the 2-*O*-benzylphosphate ligand **2,** compared to galactose. The phosphate group weakly hydrogen bonds with Arg31 while it is also stabilized by hydrogen-bonded water molecules linked to the same Arg31 and Asn51 of the CRD. The amido group also shows interactions with water molecules linked to Lys64 and Trp69, expanding the binding capacity of the ligand to a region not previously involved in galactoside recognition by galectin-7. The phenyl group does not seem to be involved in the binding of ligand **2** despite being in close proximity to polar residues His33, Glu122 and Asn35. As both His33 and Glu122 are not conserved among galectins, better specificity of inhibition could be achieved by focusing the ligand interaction toward this position. It is also noted that the benzyl moiety of the *O*-benzylphosphate **2** is not taking part in the inhibitor binding, as it faces away from the CRD. The slightly better affinity of **1** (K_d_ = 240 μM compared to K_d_ = 450 μM for **2**) toward galectin-7 reflects this; the smaller methyl group might be able to interact with Arg31, possibly via a different orientation than that of the *O*-benzyl group, and hence, show a slightly better affinity. The authors proposed to search for a more favorable interaction with Arg31 (and other nearby residues) when the (alkyl)-phosphate groups at the 2-*O* position is modified for the design of inhibitors [106].

**Figure 3:**
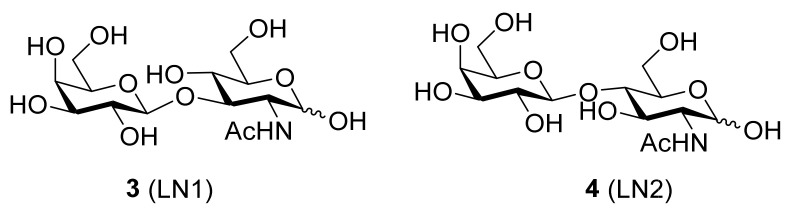
Structures of **3** (Galβ1-3GlcNAc, LN1) and **4** (Galβ1-4GlcNAc, LN2).

Hsieh and co-workers provided structural evidence of human galectin-7 (hGal7) in complex with Galβ1-3GlcNAc (LN1) and Galβ1-4GlcNAc (LN2) (Figure 3). They compared the results with LN1 and LN2-complexed galectin-1 and (the C-terminal CRD domain of) galectin-3 by means of crystallography [107].

When complexed to **3**, the crystal structure determination of galectin-7 revealed that the dimer of galectin-7 is present in a back-to-back arrangement. Furthermore, the authors confirmed that the CRD adopts a typical galectin fold, which is composed of two antiparallel β-sheets of six (S-sheets S1-S6) and five (F-sheets F1-F5) strands, jointly forming a β-sheet sandwich structure. The S1–S6 β-strands constitute a concave surface to which β-galactoside-containing glycans are bound. Generally the galactose moiety (Gal) forms more hydrogen bonds with the amino acid residues in the CRD of the galectin than the *N*-acetylglucosamine moiety (GlcNAc), supporting the idea that Gal serves as the major recognition component [107].

The Gal of **3** (LN1) interacts with the following residues located on S4–S6 β-strands and the loop connecting S4 and S5 strands of galectin-7: His49, Asn53 and Asn62 (through hydrogen bond networks) and Trp69 (via van der Waals contacts). Specifically, the Arg53 residue not only bridges H-bonds to several oxygen atoms of LN1 (C4-OH, O5 of GAL and C4-OH of GlcNAc), but also connects a few carbohydrate-interacting amino acid residues, such as Asn51, Glu58 and Arg74, to form a characteristic interacting network of H-bonds and electrostatic interactions, which are optimal for the carbohydrate orientation. Galectin-7 has more H-bonds to the Gal moiety and a characteristic shorter distance with GlcNAc in **3** (LN1), as compared to those in **4** (LN2) [107].

The electrostatic network consists of Arg53, Glu58, Glu72 and Arg74. Glu58 mediates a unique salt-bridge network by forming two weaker monodentate N–O bridges with Arg53 and Arg74. Neither of the hGal7–LN1 and the hGal7–LN2 complexes contain water-mediated interactions; the main cause is most probably the large distance of Glu58 to the bound sugar. Investigation of the loop L4 between the S4 and S5 β-strands revealed that L4 is shorter, compared to the counterpart in galectins-1 and -3. Glu58 seems to either reside in the end of L4 or the beginning of the S5 β-strand, which makes it impossible for Glu58 to coordinate with the N2 atom of LN2 for additional water-mediated interactions. Based on their results, it was concluded that the length of L4 and the location of the Glu residue (resided in the variable loop L4) are found to influence the geometry of the salt-bridge, which eventually resulted in a higher affinity of galectin-7 toward LN1, compared to LN2 [107].

**Figure 4:**
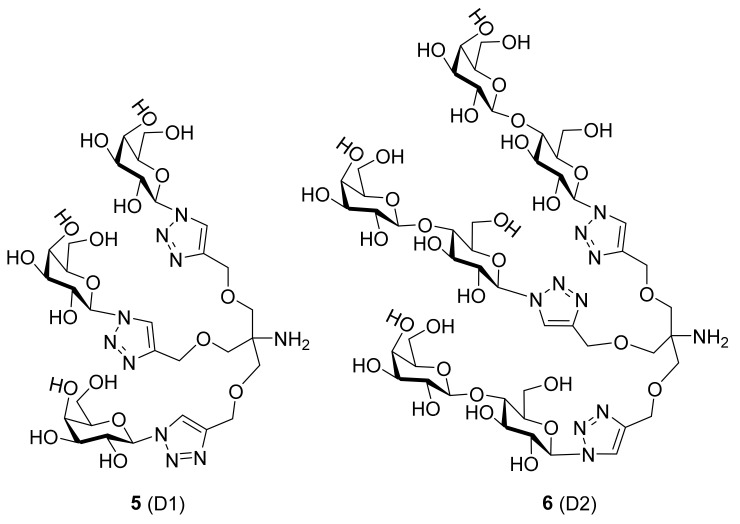

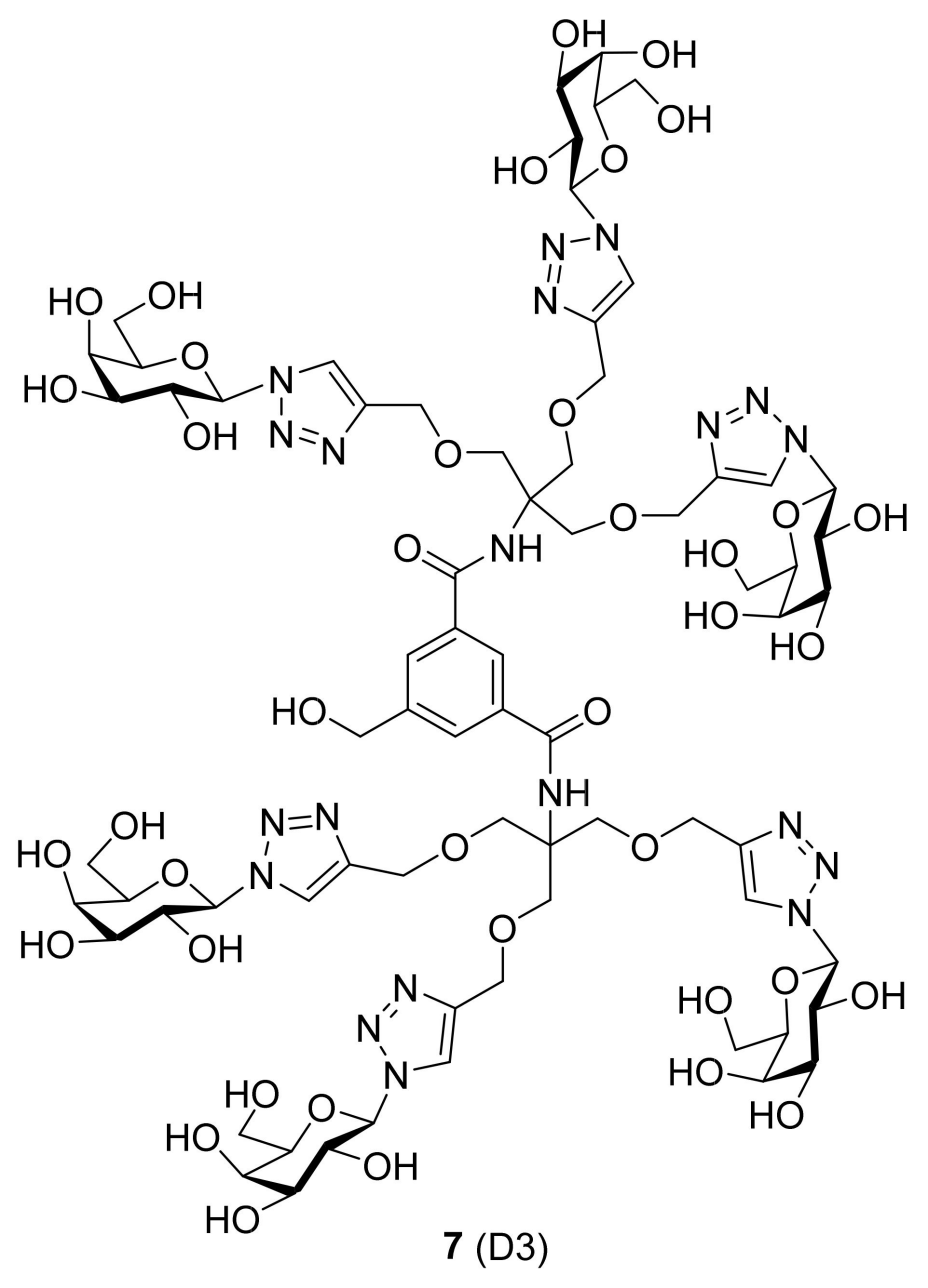
Structures of **5**, **6** and **7** (D1, D2 and D3, respectively).

High-resolution crystal structures of carbohydrate-based dendrons D1, D2 and D3 (**5**, **6** and **7,** respectively, in Figure 4) in complex with human galectin-7 were resolved, as follows. The overall structure of galectin-7 remained unchanged upon ligand binding and appeared as a dimer comparable with that described previously by Leonidas [8]. The dimeric state of galectin-7 did not appear to break down upon ligand binding; however, the interface of dimerization was slightly altered in terms of a decrease in surface contact area. The ligand D1 (**5**) is bound to galectin-7 through its galactose rings interacting with the CRD and a single water-mediated hydrogen bond between the triazole arm and R31. Despite having identical lengths, all three arms do not seem to be long enough to bind to galectin-7 simultaneously. This probably resulted in the disorder and lack of electron density for the third arm in the crystal structure of the D1–hGal7 complex. It was concluded that D1 was able to link two molecules of galectin-7 in a linear fashion as shown in Table 2. Co-crystallization of galectin-7 in complex with D2 (**6**) led to two crystal forms. The first crystal form (D2-1) showed electron density for two of the three arms of the dendrons similar to that observed with D1 (resulting in cross-linking of two hGal7 molecules). In the second crystal form, D2-2, electron density was observed for all three arms of the dendrons with each galactose-terminus bound to one hGal7 molecule; this crystal form has three dimers of hGal7 in the asymmetric unit. Galectin-7 in complex with D3 (**7**) results in the linking of two molecules of galectin-7 (Table 2). In addition, the D3–hGal7 structure of this complex shows that one terminal galactosyl group binds at the CRD of galectin-7, whereas another galactosyl ring of the adjacent arm interacts with a different CRD of the same galectin-7 molecule [108].

TD139 **8** [109], being in clinical development by the Swedish Galecto Biotech [110], has completed Phase Ib/IIa clinical trials for the treatment of idiopathic pulmonary fibrosis [111]. It displays potent inhibition of galectin-1 and galectin-3, which proved to be increased by a factor up to 200 times, compared to the inhibition of galectin-7 as determined by fluorescence polarization (FP) [112].

**Figure 5:**
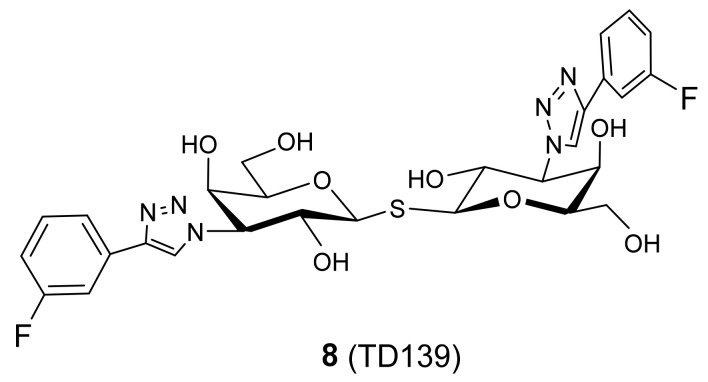
Structure of **8** (TD139).

Hsieh and co-workers investigated the binding interactions between thio-digalactoside TD139 **8** (Figure 5) with galectin-1, -3 and -7 by means of X-ray crystallography, isothermal titration calorimetry and NMR spectroscopy [113]. The galectin’s CRD is described in terms of the subsites A–E in order to facilitate analysis and discussions on ligand binding [114]. According to this model, the best structurally characterized subsites C and D are responsible for recognition of the β-galactoside–containing disaccharides [113].

When the binding affinity of 8 with human galectin-3 (hGal3) was investigated with that of human galectin-7 (hGal7), it became clear that galectin-7 contains Arg31 and His33 at the positions held by Arg144^hGal3^ and Ala146^hGal3^. Arg31^hGal7^ is placed in subsite B and thus, does not interact with the 4-fluorophenyl substituent of TD139. Likely hindered by the imidazole of His33^hGal7^, a bulkier residue than the counterpart Ala146^hGal3^, the 4-fluorophenyl moiety turns ~50° away as compared to that in the galectin-3 complex, having the vacated volume in subsite B of galectin-7 occupied by two water molecules. The orientation in which the 4-fluorophenyl-triazole moiety of TD139 is situated in subsite E (the aromatic substituent interacts with Arg) is a consequence of the previously mentioned salt-bridge in galectin-7, this time involving Glu58, Arg74 and Glu72. Similar tandem arginine–π interactions between the 4-fluorophenyl-triazole and Arg74^hGal7^ were observed, albeit being a weak interaction due to the electron-deficient π system. This π–arginine interaction resides only in subsite E (not in subsite B) as confirmed by ^19^F-NMR spectroscopy, which led to the conclusion that subsite E of galectin-7 is able to contribute more binding interactions than subsite B [113].

## 5. Small-Molecule Carbohydrate and Non-Carbohydrate Galectin-7 Inhibitors

Due to the galectin-7 characteristic that it binds to β-galactosides, most of its small-molecule inhibitors are carbohydrates, or, at least, based on sugar scaffolds. In order to make progress, it is of importance to come up with (glyco)mimetics that are capable of recognizing and blocking galectin-7. These mimetics could be molecules that mimic natural (binding) carbohydrates structurally and functionally. In addition, they should display improved pharmacological properties, have better resistance against glycosidase hydrolysis, and bind more strongly and more selectively to galectin-7 [2]. In particular, the poor selectivity of current small-molecule inhibitors remains an important obstacle to overcome, due to the high similarity of the CRD structures among the different galectins [50]. Hence, developing specific galectin-7 inhibitors that will selectively target the intracellular or extracellular functions of galectin-7 could be a strategy to inhibit not all, but specific galectin-7–mediated processes [13]. Chan and co-workers mentioned in their review that success was achieved in distinguishing between galectin-3 and other galectins. However, having the selectivity be reversed and thus developing inhibitors that are more selective for the weak-binding galectin-7 (than for galectin-3, for example) would certainly be a major breakthrough [115]. In this section, we will briefly discuss the best synthetic inhibitors of galectin-7 based on (non-)carbohydrate scaffolds that were developed in the past.

### 5.1. Inhibitors Based on a Carbohydrate Scaffold

**Figure 6:**
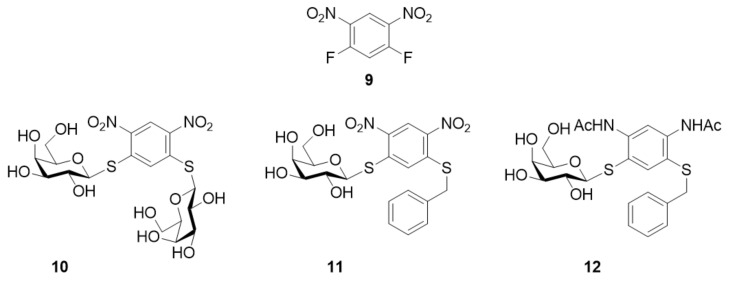
Structures of the scaffold 9 and the inhibitors **10** (K_d_ = 0.17 mM), **11** (K_d_ = 0.18 mM) and **12** (K_d_ = 0.14 mM).

The first discovery of efficient and selective monosaccharide inhibitors of galectin-7 came from the group of Nilsson during a study in which they synthesized a library of 28 compounds that was tested for binding to galectin-1, -3, -7, -8N and -9N. They demonstrated the potential of 1,5-difluoro-2,4-dinitrobenzene **9** (Figure 6) as a scaffold for the synthesis of combinatorial carbohydrate libraries. Three selective galectin-7 inhibitors (structures **10**, **11** and **12** in Figure 6) were found to have affinities similar to those of the best natural ligands. The K_d_ values were measured in a competitive fluorescence-polarization assay to be in the range of 0.14–0.18 mM for galectin-7, whereas no inhibition was observed for galectin-1, -3, -8N and -9N [116].

One year later, Bergh and co-workers from the same group published syntheses of galactosides carrying 3- or 4-substituted alkyne benzyl ethers. The group developed a method using a solid phase variant of the Nicholas reaction to provide inhibitors that have alkynyl benzyl ethers. Their approach simplified the purification steps and enabled the use of unprotected carbohydrates in the formation of the *para*/*meta*-substituted products. They found two of them to be the simple straight-chain allyl- and hydroxymethyl-substituted alkynes **13** and **14**, which suggests that the binding pocket of galectin-7 close to galactose O-3 is relatively small and does not allow larger cyclic structures to bind. The K_d_ (mM) values against galectin-1, -3, -7, -8N and -9N were measured in a competitive fluorescence polarization assay and listed in Table 3 [117]:

Compound **13** proved to be the most interesting inhibitor, due to its lowest K_d_ value and its selectivity. Compared to affinities for other members of the galectin family, preference for galectin-7 is increased by a factor of up to 100 [117].

**Figure 7:**
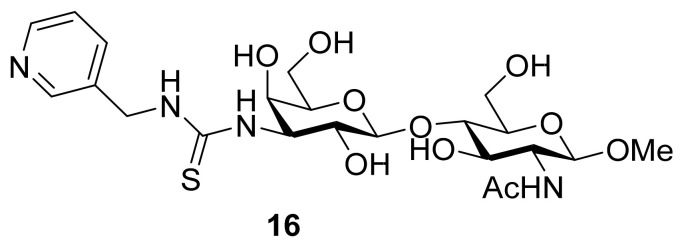
Thioureido *N*-acetyllactosamine derivative **16**.

Salameh and colleagues came up with derivatives of *N*-acetyl lactosamine carrying diverse thiourea groups at galactose C3. The thioureas obtained upon reaction of the isothiocyanate with amines are known to form strong hydrogen bonds, which makes them suitable for improving the affinity of ligands for proteins. In case of **16** (Figure 7), a K_d_ value of 23 μM was measured by a fluorescence polarization assay, which makes **16** the best galectin-7 ligand. It is, however, not the most selective, as it binds in the range of 35–47 μM to galectin-1, -3, -8N and -9N [118].

**Figure 8:**
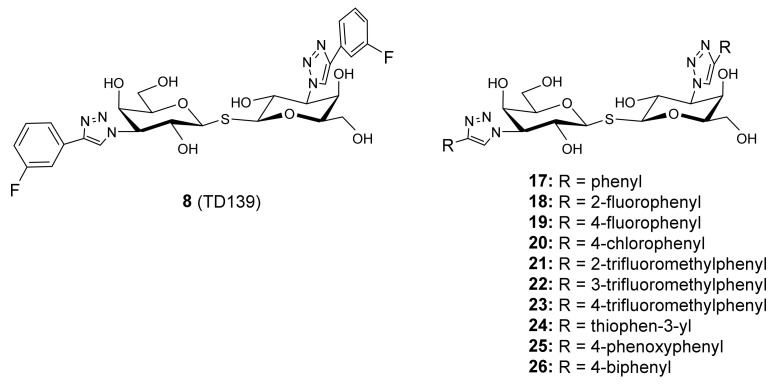
Ditriazolylthio-digalactosides developed by Delaine et al.

In a more recent paper, Delaine et al. continued developing galectin-1 and -3 antagonists with selectivity and therefore, synthesized ditriazolylthio-digalactosides (compounds **8** and **17**–**26** in Figure 8) [112]:

It was observed from the dissociation constants that, regarding the affinity of these ligands toward galectin-7, the binding is enhanced by the 4-aryltriazolyl groups in **17**–**24**. The dissociation constants are in the range of 1–10 μM for galectin-7, which are close to those for galectin-2, -4N, -4C, -9N and -9C. The sterically more demanding compounds **25** and **26** did not significantly bind to galectin-7 [112].

### 5.2. Inhibitors Based on a Non-Carbohydrate Scaffold

**Figure 9:**
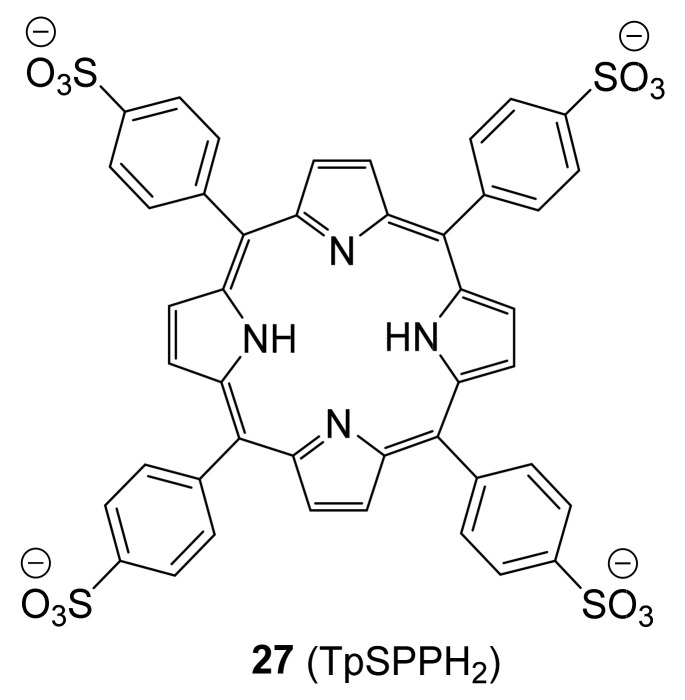
Structure of a novel non-carbohydrate galectin-7 inhibitor **27** (TpSPPH_2_).

Vladoiu and colleagues reported a peptide-based galectin inhibitor that was specifically designed to disrupt the formation of galectin-7 dimers from the monomers and its pro-apoptotic function. They identified critical residues possibly involved in the formation of the dimer interface based on their tendency to form hydrogen bonding, hydrophobic, or van der Waals interactions [8]. In addition, structural analyses of the dimeric interface published by Ermakova and co-workers [105] was also used in their design of a peptide-based inhibitor. Two peptides were designed to rationally mimic and disrupt the galectin-7 segment between residues 13–25 and 129–135 since those residues appear to be directly involved in the stabilization of the dimeric structure: hGal7_(13–25)_ (H-Ile-Arg-Pro-Gly-Thr-Val-Leu-Arg-Ile-Arg-Gly-Leu-Val-NH_2_) and hGal7_(129–135)_ (H-Leu-Asp-Ser-Val-Arg-Ile-Pro-NH_2_). Human galectin-7_129–135_ proved to be more potent than hGal7_(13–25)_ in disrupting hGal7 homodimers as measured by mild denaturing native gel electrophoresis. There is an interaction between hGal7_(129–135)_ and galectin-7 through a classical solid-phase binding assay. The decrease in hGal7 homodimers is observed at a concentration range of 100–500 μM of peptide hGal7_(129–135)_. Moreover, an increase in galectin-7 binding on the surface of Jurkat T cells and an apoptotic response were observed in the presence of hGal7_(129–135)_. A reduction in the ability of the protein to induce apoptosis of Jurkat T cells was observed [119]. More recently it was demonstrated that *meso*-tetrakis(*p*-sulfonatophenyl)porphyrin **27** (TpSPPH_2_, Figure 9) significantly reduced the level of (galectin-7-induced) apoptosis of human Jurkat T cells [120].

A binding affinity of **27** for galectin-7 was measured by fluorescence quenching to be 9.5 ± 1.6 μM. In addition, TpSPPH_2_-bridged oligomers of galectin-7 were observed by small-angle X-ray scattering (SAXS) and ^1^H−^15^N HSQC NMR of galectin-7−TpSPPH_2_ complexes. Docking simulations on galectin-7 showed that the TpSPPH_2_ moiety preferentially binds to three main subsites at the dimer interface. [120].

## 6. Conclusions

In the world of physiology, pathology and glycobiology, galectin-7 is one of many proteins that require special attention, due to its striking biological properties. Galectin-7 is a member of the prototype galectin family, which is mainly expressed in stratified epithelia of several tissues. While it is known for having multiple biological functions in the human body, much of its molecular mode of action has to be elucidated. Although several strategies were developed to knockout galectin-7 or suppress its translation, we believe the field of cellular pathophysiology would benefit from small-molecule inhibitors which can be administered to evaluate its effect on cellular disorders and even diseases such as cancer. Moreover, the use of small molecules that bind strongly and specifically to galectin-7 may also be deployed for prognosis and monitoring disorders/diseases in search of better and personalized medical treatment. Molecules were synthesized, but both the potency and specificity need to be improved. It is impossible to hit a target when our eyes are closed; therefore, with this mini review, we wish to elaborate on an emerging target within glycobiology, galectin-7.

## Figures and Tables

**Table 1:** Various roles of galectin-7 along with its modes of action.

Role	Mode of Action	References
Epidermal homeostasis of skin	Regulation of keratinocyte proliferation, differentiation and migration	[14,15,16,17,18,19,20,21,22,23,24,25]
Re-epithelialization of corneal wounds	Mediating corneal epithelial cell migration	[27]
Wound healing of PDL fibroblasts	Promoting proliferation, migration and invasion of PDL fibroblasts	[28]
Promalignant activity in gastric cancer	Lower expression levels of galectin-7 cause increase in gastric cancer cell proliferation, migration and invasion	[54]
Promalignant activity in thymic lymphoma + HNSCC	Induce MMP-9 expression	[6,55,56,64]
Increasing invasive behavior of breast cancer cells	Protecting breast cancer cells from apoptosis	[58,59,60,61]
Reducing invasive behavior of prostate cancer cells	Inhibiting motility prostate cancer cells	[63]
Pro-invasive activity in oral squamous cell carcinoma	Induce MMP-2 and MMP-9 expression	[65,66]
Protective effect on the survival of cervical squamous carcinoma patients	Inhibiting MMP-9 expression and cell invasion in cervical squamous carcinoma cells	[67,69,70]
Promoting metastasis of endometrial cancer	Reducing cell–cell adhesion and enhancing cell migration	[71]
Sensitizing bladder cancer cells to chemotherapy	Increase generation of reactive oxygen species	[72]
Negative growth regulator of neuroblastoma cells	Switch from proliferation to differentiation of cancer cells	[73]
Mediation of endometrial epithelial wound repair	Endometrial re-epithelialization is dependent on integrin mediated signaling	[80]
Abnormal placentation hence leading to the development of pre-eclampsia	Acting via the placenta to induce the systemic features of pre-eclampsia via impaired placental formation, placental inflammation and placental release of anti-angiogenic factors	[81,83]
Skin barrier impairment in keratinocytes	Protecting disruption of cell-to-cell adhesion and/or cell-to-extracellular matrix adhesion	[87]
Anti-inflammation effects, inducing autoimmune disease and transplantation rejection	Promotion, proliferation and polarization of Th1/2 cells	[88]
Causing airway structural defects, injury, and other asthma responses	Increased apoptosis occurred in bronchial epithelial cells in asthma	[89,90]
Intracellular immunity in the response against bacterial infection	Colocalizing with and surrounding group A Streptococcus (GAS, intracellular bacterium)	[91]
Accelerating allograft rejection	Up-regulation of galectin-7 expression in the allografts was directly related to T cell response	[92]

**Table 2:** Cross-linking of galectin-7 by dendrons D1, D2 and D3. Figures are re-used with permission from the copyright holder.

Compound	Cross-Linked Form
**5** (D1)	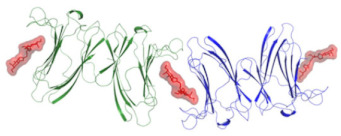
**6** (D2)	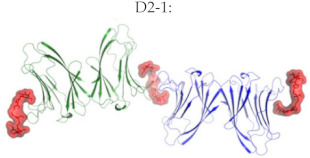
**6** (D2)	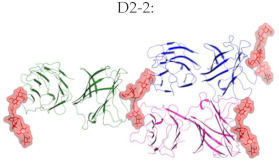
**7** (D3)	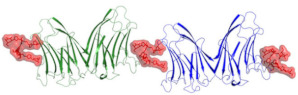

**Table 3:** K_d_ (mM) values for inhibitors **13**, **14** and **15** against galectins-1, -3, -7, -8N and -9N as measured in a competitive fluorescence-polarization assay.

Compound	Galectin-1	Galectin-3	Galectin-7	Galectin-8N	Galectin-9N
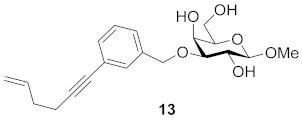	27	2.4	0.39	1.0	1.0
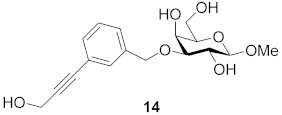	6.9	2.9	0.65	3.8	1.9
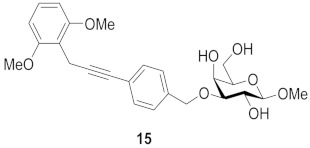	n.i. ^a^	5.4	0.74	2.4	2.0

^a^ n.i. = non-inhibitory.

## Data Availability

Not applicable.
